# Past, Present, and Future of Multisensory Wearable Technology to Monitor Sleep and Circadian Rhythms

**DOI:** 10.3389/fdgth.2021.721919

**Published:** 2021-08-16

**Authors:** Matthew R. Lujan, Ignacio Perez-Pozuelo, Michael A. Grandner

**Affiliations:** ^1^Sleep and Health Research Program, Department of Psychiatry, University of Arizona College of Medicine, Tucson, AZ, United States; ^2^School of Clinical Medicine, University of Cambridge, Cambridge, United Kingdom; ^3^Department of Medicine, The Alan Turing Institute, London, United Kingdom

**Keywords:** actigraphy, heart rate, photoplethysmography, wearables, validation

## Abstract

Movement-based sleep-wake detection devices (i.e., actigraphy devices) were first developed in the early 1970s and have repeatedly been validated against polysomnography, which is considered the “gold-standard” of sleep measurement. Indeed, they have become important tools for objectively inferring sleep in free-living conditions. Standard actigraphy devices are rooted in accelerometry to measure movement and make predictions, *via* scoring algorithms, as to whether the wearer is in a state of wakefulness or sleep. Two important developments have become incorporated in newer devices. First, additional sensors, including measures of heart rate and heart rate variability and higher resolution movement sensing through triaxial accelerometers, have been introduced to improve upon traditional, movement-based scoring algorithms. Second, these devices have transcended scientific utility and are now being manufactured and distributed to the general public. This review will provide an overview of: (1) the history of actigraphic sleep measurement, (2) the physiological underpinnings of heart rate and heart rate variability measurement in wearables, (3) the refinement and validation of both standard actigraphy and newer, multisensory devices for real-world sleep-wake detection, (4) the practical applications of actigraphy, (5) important limitations of actigraphic measurement, and lastly (6) future directions within the field.

## Introduction

The “gold-standard” measure for sleep is polysomnography (PSG). This technique includes a variety of simultaneous recordings such as electroencephalography (EEG), electromyography (EMG), electrooculography (EOG), electrocardiography (ECG), pulse oximetry, and respiration detection *via* oronasal airflow (1). PSG, *via* its measurement of physiologic changes, including measurement of brain waves from EEG electrodes, is utilized for measurement and classification of various sleep stages such as non-rapid eye movement (NREM) and rapid eye movement (REM) sleep. This measure, alongside the other physiologic channels, is one of the major reasons that PSG measurement is so valuable for sleep science and medicine. It is not without limitations, though. For example, sleep staging relies on both spatial and temporal averaging of background electrical activity and therefore cannot directly assess subcortical structures (from which sleep-wake regulation is generated, like the thalamic reticular nucleus) (2), nor can it resolve action potentials, or local wakefulness in small cortical areas, or influences of waveforms that fall outside of those used to score stages (3). For these reasons and others, PSG is an indirect and imperfect measure of sleep. Further complicating this issue is that PSG requires human scoring of raw signals; automated scoring is still not accepted, and it is unusual for 2 human scorers to achieve 100% agreement on any sleep record. Therefore, regardless of the degree of precision and/or accuracy achieved by PSG, it has inherent flaws with reliability as a gold standard, as even the same record will be scored differently by 2 raters (4).

In addition to these limitations, PSG is still quite burdensome and expensive, limiting assessment at scale and ability to assess real-world sleep parameters. On average one night of PSG measurement in a sleep lab can cost up to $2,000 dollars (5), due to the price of the equipment as well as the study personnel who monitor the individual over the night. This of course means that PSG is impractical for long-term, longitudinal studies due to the hefty price tag. To further compound this issue, there exists a well-documented “first night effect” (6–8). Due to the number of wires and electrodes strapped to a subject as well as sleeping in a new unusual environment, this has been shown to lead to increased sleep latency, decreased sleep efficiency, and decreased REM sleep.

**Figure 1:**
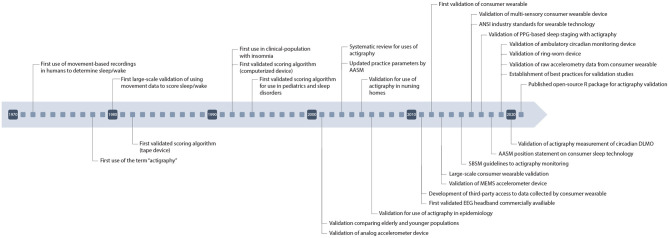
Timeline of major developments in wearable technology.

To combat these problems, researchers instead utilize movement-based devices known as “actigraphs” which measure changes in motion of an individual to predict sleep vs. wake (9). Actigraphy is an affordable alternative to PSG that can be used in free-living conditions to objectively measure sleep longitudinally as well as imposing less of a burden on subjects themselves. Actigraphy devices measure global data, including movement, to estimate sleep timing. These devices typically employ accelerometers which sample multiple axes at high frequencies to detect movement. Newer, commercial devices also house accelerometers but also include additional channels allowing them to be classified as multi-sensory and become more accurate in their scoring abilities (10). These newer wearable devices have also been able to predict sleep stages thereby giving additional scoping to their utilization for research purposes. These devices can be used to measure common sleep variables such as total sleep time, sleep efficiency, and wake after sleep onset. Figure 1 depicts many of the historical milestones relating to actigraphy and related technologies.

## History of Actigraphic Sleep Measurement

In the early 1970s, the first utilization of telemetric mobility data to estimate sleep and wake were recorded by Foster et al. (11) and Kupfer et al. (12). These studies investigated a sample of psychiatric patients to research the notion that movement data allowed for predictions of an individual's sleep timing. The devices used were rather rudimentary but still yielded an accurate representation of sleep and wakefulness over a 24-h period. Use of the lightweight, wrist-based telemetric devices was desirable due to the impracticality of using EEG-based studies for 24-h monitoring.

Kripke et al. reported similar accuracy at determining sleep-wake patterns in free-living conditions using these devices and were the first study to refer to this technique as “actigraphy” (13). The device used by Kripke et al. was an improvement of the original devices, allowing more flexibility to the wearer and utilized piezoelectric transducers, which later became the standard for all movement-based measurement. The technology enabled for more sensitivity and overall improved scoring of wake and sleep. These advances were further validated by Mullaney et al. in the first larger-scale validation study (14). This study incorporated a larger number of subjects (*N* = 102, vs. 8 and 5 in the previous studies), some of which had various sleep disturbances. Notably, the researchers were unable to demonstrate strong performance of actigraphy in older individuals and in those with sleep disturbances. The investigators also proposed the possibility of automated scoring of actigraphic data to improve scoring reliability and standardize the implementation of actigraphy overall. Through these early findings, the investigators advocated for the continued use and development of actigraphic devices due to its much lower cost compared to EEG-based studies.

As previously mentioned, PSG requires hand scoring of raw data with notable disagreement between scorers of the same data (4). There has been some success with automated scoring for PSG including a study published by Malhotra et al. which demonstrated similar results compared to that done manually (15). Yet, automated scoring for PSG is not fully accepted as the field still relies on manual scoring. For actigraphic data, the first automated scoring algorithm to demonstrate validity relative to PSG was implemented by Webster et al. (16). The development of Webster's algorithm was based on the direct comparison of actigraphy against PSG recordings. Additionally, instead of the 30-s epochs from gold-standard PSG, the algorithm used 1-min epochs to score sleep/wake due to memory limitations of the device. Several iterations of the algorithm were proposed using weighted sums of the epochs to accurately determine wake from sleep. Notably, the algorithms were more likely to misscore wake as sleep than misscore sleep as wake—a problem that continues to exist in modern actigraphy-based sleep/wake classification models (17). By demonstrating a rate of agreement between PSG and actigraphy of over 90%, the work conducted by Webster et al. established the standard for future scoring algorithms to follow.

The next cornerstone actigraphy algorithm to be published was described by Cole et al. (18). This group sought to improve upon Webster's findings by applying the principles to determine if they were generalizable to newer, commercial micro-computerized actigraphic devices (vs. the older, tape-based devices). Their final algorithm demonstrated an 88.25% accuracy compared to PSG. When the group applied Webster's original algorithm to the same data set, they found to accuracy to be slightly lower at 87.73%. This finding further validated Webster's algorithm scoring rules. The next iteration of actigraphy-based algorithms came a few years later, through the work of Sadeh et al. (19). The study conducted by Sadeh et al. not only aimed to develop a new means of automatic scoring but also address the lack of pediatric and sleep-disordered patients in previous validation efforts. The group was able to record a >91% accuracy between actigraphy and PSG scoring. Sadeh identified large differences between various devices but the relative scoring of sleep/wake was maintained despite these differences.

These early validation efforts focused on correlation coefficients and accuracy alone as the measurements for the utility of a given device. However, attention needs to be given to measures of sensitivity and specificity in addition to accuracy for validation (20, 21). These measures are derived as a function of PSG ground-truth, with accuracy corresponding with the overall ability of a device to correctly classify epochs as either sleep or wake, sensitivity with correctly classifying sleep epochs, and specificity with correctly classifying wake epochs. Actigraphic devices, and their respective scoring algorithms, are optimized to record nighttime sleep and thus have very high sensitivity and will correctly predict nearly all sleep epochs, but they have moderate to poor specificity leading to misidentification of wake as sleep. Recently, Roberts et al. demonstrated this challenge to accuracy by utilizing a naïve model (10). In this model, every epoch was scored as sleep and was marked with an 88% accuracy. Because most of a sleep record is in fact sleep, these devices are relatively accurate overall due to their high sensitivity, but poor specificity means they will likely overestimate total sleep time.

**Figure 2:**
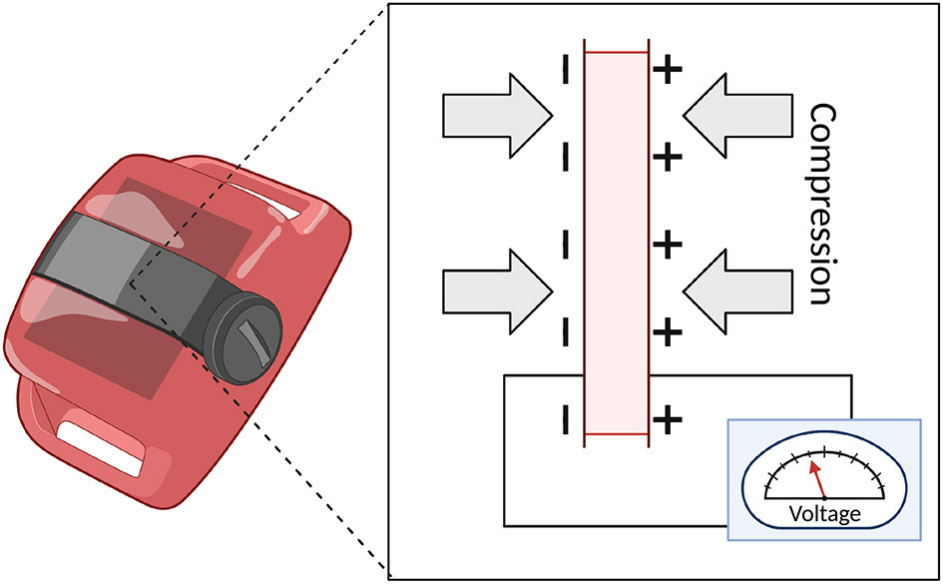
Schematic of Piezoelectric accelerometry.

The next iteration of the technology was represented by accelerometry. Early devices employed a multi-directional analog sensor and tape recorder to collect data (22). These types of devices utilize piezoelectric accelerometers that convert changes in pressure due to acceleration (i.e., movement) into changes in voltage. See Figure 2. Essentially, these devices contain piezoelectric materials, such as crystals or ceramics, that respond to the force displacement (or compression) of a mass and convert the physical energy into electrical energy.

The application of these accelerometers was practically implemented into wrist worn devices which would measure the accelerations across the radius-to-ulna axis (23). Notably, the earlier devices relied on reaching a threshold to denote a movement as activity. As such, these devices were less sensitive and could fail to capture small movements such as those occurring during sleep.

A study conducted by Terrill et al. aimed at improving actigraphy by allowing for the storage of raw sensor data instead of an activity score for each epoch (24). This group also sought to record accelerometry data tri-axially with the purpose of increasing the capacity to record more types of movements. These findings were soon followed by the application of developments in nanotechnology to existing accelerometry techniques. These developments took shape in microelectromechanical systems (MEMS) which are like the accelerometer utilized by the Terrill study. Instead of recording a single count for each desired epoch, MEMS accelerometers can yield long-term recording of raw tri-axial accelerometry at high samples rates (25). This allows for greater sensitivity and less variability between different devices in terms of raw data captured.

In addition to changing hardware and devices, there is a shift in how to utilize the captured data with the newest systems incorporating AI and machine learning techniques for sleep scoring and classification to improve accuracy. These techniques work by undergoing a training phase and subsequently learning and optimizing the model to best suit the desired analysis (26). The process for machine learning analyses requires a time-consuming and often expensive design but holds great promise for improving sleep/wake scoring and overall better sleep staging. Deep learning methods also allow for fluid processing of the data captured by many of the modern, multisensory devices currently available. In a recent study, Haghayegh et al. directly tested deep learning against standard algorithms (27). The group found that compared to algorithms, the novel deep learning model yielded greater accuracy and specificity with a lower sensitivity. This study indicates that the next generation of sleep scoring methods will be rooted in these techniques.

## Overview of Standard Actigraphic Devices

Actigraphic devices have been validated over the years for usage in determining sleep/wake timing. These validation studies compare the actigraphy data to the gold standard PSG devices to assess the relative accuracy of the device. Typically, these studies compare devices by examining sleep parameters, such as total sleep time (TST), sleep latency (SL), wake after sleep onset (WASO). Using the algorithmic developments that were previously mentioned, these devices can estimate these relative sleep parameters (28). The processing methodologies vary depending on the device and the algorithm utilized to process the data into activity counts. These activity counts are then codified as sleep or wake which can then be used to calculate the various sleep intervals. These studies also utilize epoch-by-epoch (EBE) analyses of sensitivity, specificity, and overall agreement to compare performance to differentiate sleep from wake and, more recently, differentiate estimations of the various EEG-derived sleep stages.

### Validation of Standard Devices Against PSG

Standard actigraphy devices have been readily validated against PSG for their use in objective sleep estimation (17, 29–31). These devices generally tend to overestimate certain sleep parameters such as TST and sleep efficiency (SE) (relative to PSG) and are often utilized for longitudinal data collection where expensive PSG measurement would be unfeasible or inappropriate (9).

In a study conducted by Paquet et al., four different algorithms were used in conjunction with the Actiwatch-L (Mini-Mitter, later acquired by Philips Respironics) and indicated that the device significantly underestimated sleep latency across all conditions (17). Additionally, the device tended to overestimate TST and SE, relative to PSG. This trend was more pronounced in sleep records that contained more wakefulness and on average. For EBE analysis, the device had around 90% accuracy, sensitivity around 91%, and specificity around 50%.

The Actiwatch-L was again tested against PSG and yielded modest improvements. Rupp et al. found that the updated device by Mini-Mitter underestimated TST and SE relative to PSG, overestimated the number of awakenings relative to PSG, and generally underestimated SL relative to PSG (29). For EBE analysis, the device had an accuracy around 89%, sensitivity around 92%, and specificity around 54%, compared to PSG. This group also tested the Basic Mini-Motionlogger (Ambulatory Monitoring) to find that the device underestimated TST and SE relative to PSG (29). The EBE analysis revealed the device had an accuracy of 93%, sensitivity of 96%, and specificity of 63%, compared to PSG.

Zinkhan et al. tested the validity of the ActiGraph GT3X+ (ActiGraph). The device overestimated TST and SE relative to PSG while underestimating sleep onset latency (SOL), WASO, and number of awakenings after sleep onset (NASO) relative to PSG (30). These results were followed up by a study by Quante et al. which tested different algorithms on the raw data captured by the ActiGraph GT3X+ device (31). The Sadeh algorithm led to underestimated TST and SE and overestimated WASO. The Cole-Kripke algorithm led to overestimated TST and SE and underestimated WASO relative to PSG. For EBE analysis, the device showed accuracy 83–85%, sensitivity 91–96%, and specificity 35–47%, compared to PSG.

Quante et al. tested the Actiwatch Spectrum device. The device overestimated TST and SE while underestimating WASO relative to PSG (31). For EBE analysis, the device was shown to have an accuracy of 84%, sensitivity of 95%, and specificity of 34%, compared to PSG.

**Figure 3:**
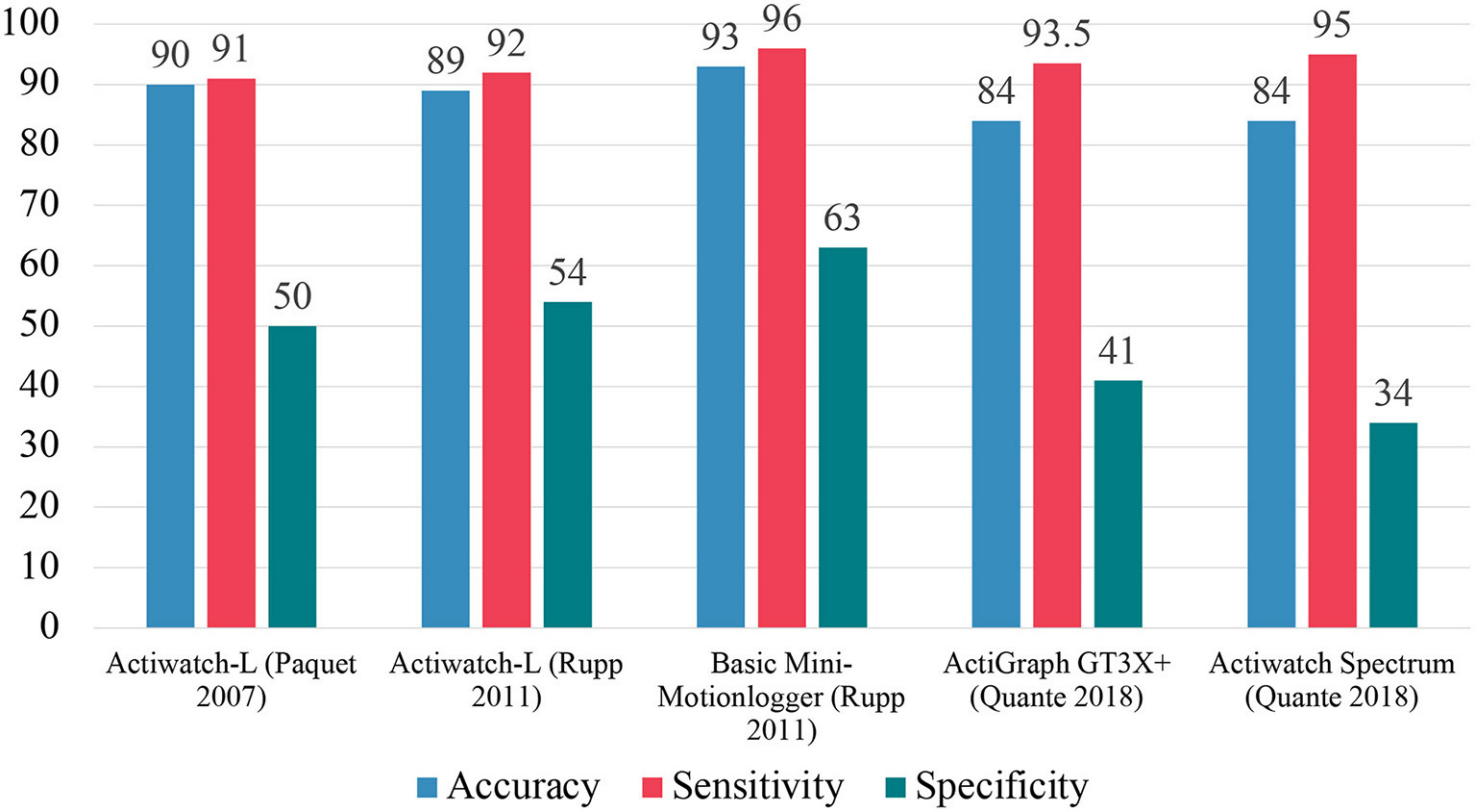
Summary of EBE analysis of standard actigraphic devices. Accuracy measures the overall ability of a device to correctly classify epochs as either sleep or wake, sensitivity measures ability to classify sleep epochs, and specificity measures ability to correctly classify wake epochs.

These findings (summarized above in Figure 3) indicate that these devices have a high level of accuracy and sensitivity. However, there exists a large variance across specificity with most devices yielding moderate agreements compared to PSG. This phenomenon exists because both devices and scoring algorithms are optimized to measure nighttime sleep. Yet these devices are relatively accurate overall due to their emphasis on sensitivity. With their poor specificity means the likelihood to overestimate total sleep time. Therefore, attention to improving the specificity (wake detection) of these devices is warranted.

## Overview of Next-Generation Wearables

Newer, multisensory devices have shown a great rate of adoption, with devices like Fitbit trackers, Apple Watches, and Oura Ring devices having a strong presence in the consumer market (32). These devices notably also record aspects of cardiac function in addition to sleep and utilize heart-rate, and sometimes heart-rate variability, assessments in sleep-wake estimation and even multi-stage sleep classification. Including these metrics offers the potential for improvements in the specificity captured by these devices.

### Anatomical and Physiologic Basis of Using HR

Mechanistically, the heart works as a double pump with the right side and left side of the heart working in anatomically separate circulations (33). The right-side pumps to the lungs whereas the left side pumps out to the body. Diastole is the relaxation and filling period of a cardiac cycle whereas systole represents the contraction and ejection period. During diastole, the pressure in the heart chambers is low, as the volume in the ventricles increases over time. During systole, the pressure in the heart chambers is increasing as the heart squeezes and contracts to eject the pooled volume. These changes in volume and pressure are particularly relevant for measurement at the periphery.

**Figure 4:**
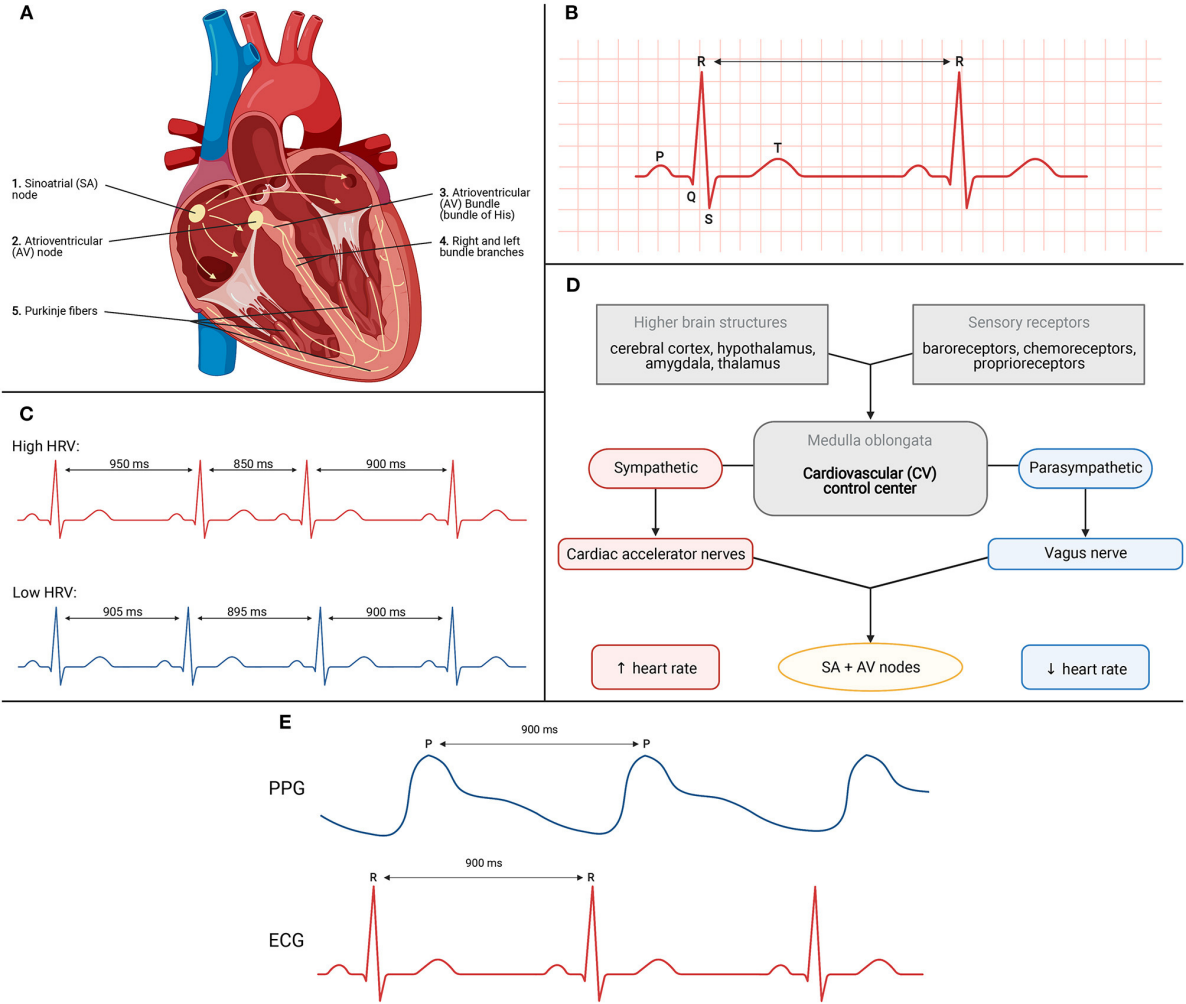
**(A)** Conduction system of the heart. **(B)** Schematic for ECG recording of electrical activity. **(C)** High vs. low heart rate variability (HRV). **(D)** Schematic depicting various factors influencing HR and HRV. **(E)** Correlation between R-R interval from ECG trace to P-P interval from PPG trace.

These mechanical, physical movements are preceded by electrical activity priming the heart. The gold standard technique for assessment of electrical activity of the heart is the electrocardiogram or ECG. ECG recording measures voltage changes over time which underly the conduction system of the heart (34). The electrical process and action potential is initiated at the sinoatrial (SA) node. This structure is typically referred to as the pacemaker of the heart. These action potentials then travel to the atrioventricular (AV) node which notably delays the impulse briefly to allow sufficient time for diastole. From here, the impulse travels to the AV bundle which electrically connects the atria and the ventricles. And finally, ending the conduction system is the right and left bundle branches and the Purkinje fibers. The propagation of electrical signals allows for efficient, coordinated contraction of the heart. A schematic of this process is depicted in Figure 4A.

The conduction system generates action potentials which can be visually interpreted as the waveforms and segments on the ECG recording (34). See Figure 4B. The three major waveforms are the P wave, the QRS complex, and the T wave. The P wave coincides with atrial depolarization, the QRS complex with ventricular depolarization, and the T wave with ventricular repolarization. Atrial depolarization leads to atrial contraction, ventricular depolarization leads to ventricular contraction, and ventricular repolarization leads to ventricular relaxation. Also important to the utility of the ECG trace is observation of the R-R interval. This is indicated by the arrow between two consecutive QRS complexes. The R-R interval is the time between adjacent R waves and is used to estimate an individual's heart rate, or number of beats per minute (34). Inclusion of the ECG during a sleep measurement period allows researchers to monitor changes in cardiac function over time to correlate with measures of sleep vs. wakefulness.

### Heart Rate Variability

Heart rate variability (HRV) measures the variance in R-R interval timing. HRV can be used as a biomarker as high or low values have been correlated to different physiological states. A high heart rate variability, indicated in Figure 4C with the red ECG trace, shows highly varied R-R intervals over time. This high variance is indicative of parasympathetic tone, demonstrating that the body has a higher capacity for stress and adaptation, and indicates good fitness of the individual (35). A low heart rate variability, indicated with the blue ECG trace in Figure 4C, shows little to no variation across R-R intervals. This low variance indicates factors such as high sympathetic tone, low adaptability to changing environments, and acts as a sign of bodily stress, such as during exercise.

HRV is rather dynamic with different factors mediating its changes over time (36). The inputs and outputs to this feedback mechanism are depicted in Figure 4D. They are housed within the medulla oblongata of the brainstem which receives sensory information from various components. Based on the change required, the control center outputs to the heart *via* the sympathetic or parasympathetic nervous system. The sympathetic effects will increase rates of depolarization to increase heart rate whereas the parasympathetic effects accomplish the opposite by decreasing rates of depolarization leading to decreases in heart rate. As these changes are occurring in real time and from beat-to-beat, measuring HRV yields an indirect measure of the underlying autonomic control which can be extrapolated to measures of sleep vs. wake.

### Wearable Measurement of HR and HRV

Photoplethysmography (PPG) is an optical technique that can be used to measure changes in blood volume and pressure at the periphery (37). The two major components that comprise a wrist-worn PPG sensor are (1) an LED light and (2) a photodetector (38). This set-up is known as reflective PPG. The light emitted by the LED penetrates the skin to the level of capillaries. There the light is both absorbed and reflected. The light that is reflected is received by the photodetector. These sensors process and quantify changes in reflectance to coincide with changes in blood volume and pressure which have been validated to show their accuracy in measuring heart rate during both periods of rest and activity (39).

PPG sensor technology varies across devices in the type or wavelength of light emitted from the LED. Wearable devices typically use green light (40), which has a comparatively shorter wavelength compared to red or infrared light. The shorter wavelength light contains a greater amount of energy and thus is better able to penetrate the skin. The light is readily reflected by vessels closer to the surface and thus is less influenced by blood flow in deeper vascular networks and subject to less noise effects (40). This in turn leads to green light PPG estimating pulse rates that are strongly correlated with the R-R interval from the ECG (40).

But how does measuring reflectance changes at the wrist measure heart rate (HR)? When the heart beats during systole, blood flows out of the left side of the heart and all throughout the body including to the periphery such as the wrist. This increase in blood volume results in more green light absorbed and therefore less reflectance picked up by the PPG sensors (40). In contrast, between heart beats and during diastole, less blood is flowing out to the periphery. As less blood volume is present, less green light is absorbed leading to more reflectance picked up by the PPG sensor. Wearable devices flash their LEDs at extremely high frequencies to quantify and measure the changes in reflectance (41). These measurements are then processed to correlate with heart rate. Figure 4E depicts the relationship between the RR interval from the ECG trace and the peak-to-peak (PP) interval from the PPG trace. The PP interval represents increased pressure at the periphery following systole. Due to the brief time required for blood to flow out to the periphery, the RR interval and PP interval appear to have a delay but are phase aligned as observed in Figure 4E. In other words, the timing of each interval is identical and allows PPG to be utilized for indirect measurement of heart rate *via* RR intervals.

However, the measurement of PP intervals *via* PPG is not a complete substitute for RR intervals *via* ECG. Therefore, researchers should take caution in using PP intervals as a proxy for RR intervals as it is not accurate in all individuals (42). It has been reported that adults with pacemaker devices show fluctuations in PP intervals without accompanying fluctuations in RR intervals. This exists due to the downstream nature of the PP interval. Yuda et al. recommend that PP intervals be considered a separate biomarker but recognize that HRV has a significant influence on changes in blood flow at the periphery. If researchers are consistent and aware of these issues, the utilization of PPG is suitable for indirect monitoring of changes in cardiovascular function.

### Correlation of HR and HRV to Sleep and Sleep Staging

Snyder et al. showed that heart rate decreases progressively over the course of the night (43). HR decreases specifically as an individual enters deeper stages of sleep. It is highest during wake and high during REM sleep but decreases subsequently from Stage 1 onward. These trends are consistently found across individuals and can be used to improve the accuracy of sleep/wake classification by scoring algorithms. Additionally, HR spikes with awakenings thereby giving rise to potential means to improve specificity (wake) measurement. Other abnormalities in HR during sleep have been demonstrated to underly certain disease states such as obstructive sleep apnea (44, 45).

But heart rate alone is not sufficient to get an accurate estimate of cortical sleep stages. Indeed, several studies have tried using heart rate variability to enhance the performance of models (46–49). The frequency-domain perspective is well-correlated with the various sleep stages (46, 50, 51). Using spectral analysis techniques, the specific frequency bands give insight into the relative parasympathetic to sympathetic control. Very low frequency (VLF) variability is seen at 0.00–0.05 Hz, Low frequency (LF) variability is 0.05–0.15 Hz, High frequency (HF) variability at 0.15–0.40 Hz, and finally with total power encompassing the entire spectrum from 0.00 to 0.05 Hz.

Correlations between sleep stages and HR frequency bands are typically normalized to total power, because total power itself varies by time of day. Therefore, relative power is typically assessed to quantify the effects of changes in total spectrum power. VLF generally yields the measure of sympathetic influences and is seen highest during REM sleep. LF is a mixture of both sympathetic and parasympathetic influences with the band lowest during Stage 3 NREM sleep. HF suggests parasympathetic influences and generally is increased in NREM sleep and decreased in REM sleep. Specifically, this frequency band is significantly higher during Stage 2 NREM sleep. Total power is shown to be lowest during wake and highest in REM sleep.

These correlations (summarized in Table 1), as well as the general patterns observed for HR during sleep measurement, offer the potential for improved sleep staging as well as sleep/wake scoring in general with particular emphasis on improving specificity.

**Table 1:** Summary chart of correlations of HRV frequency bands to sleep stages.

	**Frequency (Hz)**	**ANS indications**	**Correlated sleep stage**
Very low frequency (VLF)	0.00–0.05	Sympathetic tone	Highest in REM
Low frequency (LF)	0.05–0.15	Sympathovagal balance	Lowest in stage 3 NREM
High frequency (HF)	0.15–0.40	Parasympathetic tone	Highest in stage 2 NREM
Total Power	0.00–0.40	–	Lowest in wake; highest in REM

### Validation of Devices That Use Heart Rate

Due to the valuable information that heart rate and other cardiovascular variables may contain, the desire to have implemented heart rate and heart rate variability sensors into devices has arisen. This inclusion allows for better scoring of sleep/wake and predictions for sleep staging/architecture. These devices include well-known brands such as Fitbit, WHOOP, Apple Watch, Garmin, Polar, and Oura. While the market is quite diverse and saturated with many different products, this review will focus on the validation of Fitbit, Apple Watch, and Oura Ring devices.

#### Fitbit

There are many Fitbit devices on the market. Most of the devices produced in the past several years are equipped with both an accelerometer and an optical PPG sensor (52). However, older devices contained only an accelerometer to estimate sleep based on movement alone. Regarding the devices with both movement and heart rate assessment, the technology used to assess sleep vs. wake is essentially identical across devices (53).

Montgomery-Downs et al. investigated the validity of the original Fitbit Tracker as a tool to classify sleep/wake (54). This device was equipped only with a tri-axial accelerometer and no sensor to collect data on heart rate variables. The device overestimated both TST and SE relative to PSG. For EBE analysis, the device showed a sensitivity of 97.8% and specificity of 19.8%.

The first Fitbit device that added a PPG sensor and underwent validation was the FitbitChargeHR. In the study by de Zambotti et al., the FitbitChargeHR overestimated TST and SE while underestimating WASO relative to PSG (55). The device was found to have a sensitivity of 97% and specificity of 42%. de Zambotti et al. later assessed the Fitbit Charge 2 in terms of measuring sleep/wake as well as “light” (PSG N1+N2), “deep” (PSG N3) and REM sleep (56). This device also included a PPG sensor for the collection of heart rate variables. The device overestimated light sleep and underestimated deep sleep, relative to PSG. In EBE analysis, the device showed a sensitivity of 96% and specificity of 61%. For individual sleep stages, the device had an accuracy of 81% for identifying “light” sleep, 49% for identifying “deep” sleep, and 47% for identifying REM sleep.

The large-scale Fitbit validation study conducted by Beattie et al. assessed the Fitbit Surge device (57). The device had an overall sensitivity of 94.6% and specificity of 69.3%. The device also showed a 69.2% agreement with PSG measured “light” sleep (stage N1 and N2 combined), a 62.4% agreement with PSG measured “deep” sleep (stage N3), and a 71.6% agreement with PSG measured REM sleep. This study also demonstrated that the device may be more effective in measuring some people than others. Best performance was shown in individuals with consolidated sleep with less WASO.

A recent study conducted by Chinoy et al. assessed the Fitbit Alta HR device (58). The device showed an overall 90% accuracy, sensitivity of 95%, and specificity of 54%. For individual sleep stages, the Fitbit Alta HR had an accuracy of 72% for classifying “light” sleep, an accuracy of 86% for classifying “deep” sleep, and an accuracy of 89% for classifying REM sleep, all relative to PSG.

#### Apple Watch

Apple Watch devices are equipped with a few different sensors. Notably, these include an ambient light sensor, multiaxial accelerometer, and optical plethysmography sensor (59). These devices use proprietary algorithms to determine relevant movement and heart rate data, with newer devices enabling proxy measurements of ECG rhythms and blood oxygen concentration.

The Apple Watch, until the latest Series 6 device, did not natively have a sleep/wake scoring function that is comparable to other devices, but with its available sensors the data can be recorded and subsequently scored using various analyses. Walch et al. investigated the Apple Watch Series 2 and 3 to investigate the validity of these devices to be used for sleep monitoring (60). The group extracted raw accelerometry data captured by the MEMS accelerometer and heart rate data from the PPG sensor. Since the ambient light data is not accessible for analysis, a “clock proxy” was also introduced to include an element of circadian timing to improve scoring. The circadian clock proxy was generated in two different ways. The first, used a fixed cosine wave from the start of recording to simulate circadian rhythms. The second, was generated using the recorded step data and converted on a scale that coincided the movement recording, if above a certain threshold, with the approximate lux depending on the time of day. The best model utilized neural net methods and the combination of accelerometry, heart rate, and circadian data. Compared to PSG, the model yielded an accuracy of 90.1%, sensitivity of 93%, specificity of 59.6% with a kappa of 0.449. For sleep staging, the neural net model with all three features led to 60% accuracy in detecting wake epochs, 65.1% accuracy in detecting NREM epochs, and 65% accuracy in detecting REM epochs, relative to PSG.

These devices were further investigated by Roberts et al. by utilizing machine learning techniques (10). The group extracted the accelerometry data and PPG heart rate data for implementation into scoring techniques. Notably, the PPG heart rate data was transformed into pseudo-interbeat intervals values to allow for comparison across different devices and manufacturers. The greatest machine learning analysis of the Apple Watch data collected yielded an accuracy of 93%, sensitivity of 98%, specificity of 60% with a kappa of 0.602. These two studies showed that the Apple Watch is capable of sufficiently scoring sleep/wake based on the data captured.

#### Oura Ring

The OURA ring collects data on pulse rate, HRV, respiratory rate, body temperature, and nighttime movement (61). The Oura Ring is notably different from most wearables including the Fitbit and Apple Watch because it is worn on the ring finger of an individual rather than the wrist. Because of this difference it is worth discussing the differences in measuring HR at the finger before looking at validation data.

In a study conducted by Longmore et al., investigators utilized both red and infrared-based PPG sensors and measured the error rate compared to ECG (62). It was discovered that HR measurement at the finger was less prone to error than HR measurement at the wrist. The investigators postulated that this is due to the wrist having more artifact disruption due to ligaments and underlying tissues that interfere with adequate readings.

The validation study conducted by de Zambotti et al. investigated the potential of this device to be used in the context of sleep/wake scoring and classification of individual sleep stages (63). Notably there were no significant differences between the sleep summary variables SL, TST, and WASO as compared to PSG. In EBE analysis, the device showed 96% sensitivity and 48% specificity. For individual sleep stages, the device had 65% agreement in classifying “light sleep,” 51% agreement in classifying “deep sleep,” and 61% agreement in classifying REM sleep, as compared to PSG.

The validation study conducted by Roberts et al. also investigated the OURA ring (10). The device had good agreement with PSG in terms of estimates of WASO, TST, and SE. In EBE analysis, the device showed 89.9% accuracy, 96.3% sensitivity, 41.0% specificity, and kappa of 0.423. Normalization *via* machine learning analyses were applied to the data for additional scoring and this process led to minor changes in EBE measures; 90.6% accuracy, 97.1% sensitivity, 40.7% specificity, and kappa of 0.418. Oversampling of wake epochs during the training phase improved specificity to 70.7% while reducing sensitivity to 85.3%.

### Comparisons to Standard Actigraphy

Consumer wearables, including Fitbit, Apple Watch, and Oura Ring, include more sensors (i.e., use of PPG in addition to accelerometry), which allows for a greater opportunity to achieve accurate sleep/wake scoring. With these improvements, these devices may better serve clinical populations, such as those with insomnia, based on increased specificity (wake detection). These consumer devices are also generally less expensive and more available than some of the older devices. As opposed to standard actigraphic devices, these consumer wearables can modestly predict sleep stages because of their inclusion of other sensors. Regarding weaknesses, the scoring algorithms utilized by these consumer devices are typically proprietary to the developers with little access to raw data. Second, because these devices are commonly updated with new hardware, firmware, and software updates, it is not clear the degree to which any specific iteration alters accuracy or other aspects of performance in context. For example, an update could either increase or decrease the accuracy of the device. This would be especially detrimental if such an update occurred during an ongoing study.

Even a standard actigraph when combined with ECG and HR data leads to improved performance (64). In this secondary analysis conducted by Zhai et al., the MESA sleep study data, including actigraphy data from an Actiwatch Spectrum (Phillips Respironics) and ECG data from a Compumedics Somte System for PSG, was extracted. The extracted movement and cardiac data were used to generate models and algorithms to evaluate the dataset. The results of the analysis showed that neural network models outperform traditional machine learning and heuristic methods for both scoring sleep vs. wake and estimation of sleep stages. Additionally, with an ensemble method to estimating sleep stages, the group was able to yield an accuracy of 78.2% for three sleep stages (Wake, REM, NREM) and 65.4% accuracy for the five individual sleep stages (Wake, REM, N1, N2, N3). This study was significant as it demonstrated that a multimodal approach could be utilized for accurate sleep measurement.

**Figure 5:**
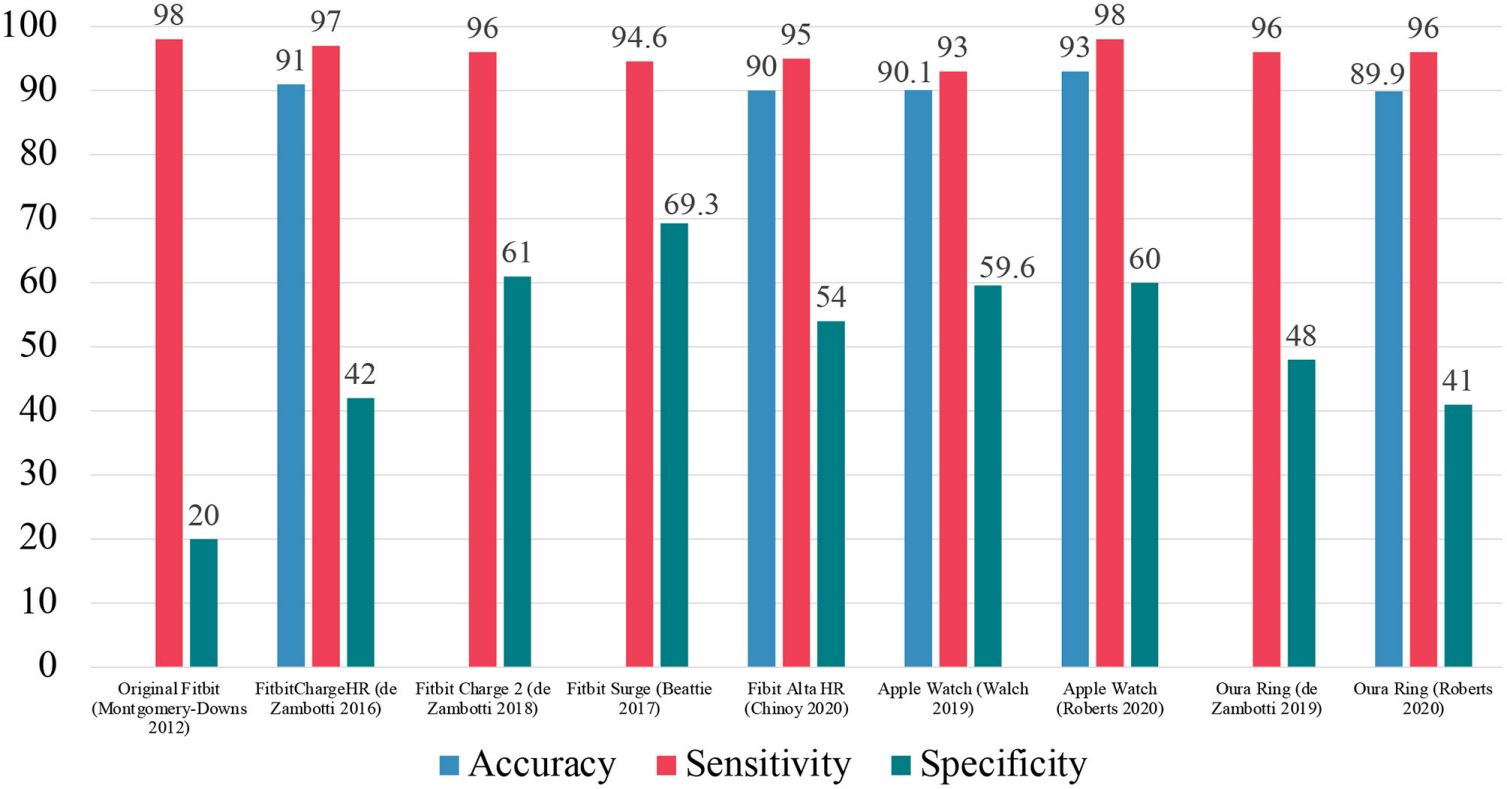
Summary of EBE analysis for Fitbit, Apple Watch, and Oura Ring devices.

Figure 5 summarizes the EBE analysis for the validation studies for Fitbit, Apple Watch, and Oura Ring devices. Importantly, it should be noted that the Fitbit Charge 2 and Apple Watch devices performed as well or better than the standard actigraphs included in Figure 3.

## Other Categories of Consumer Sleep Technology

### Phone-Based Accelerometers

As smartphones have become ubiquitous, app developers have leveraged the built-in accelerometers to record movement data during sleep periods (65). The general assumption is less movement equates with the transition from light to deep sleep. Two of the most popular apps that record movement data are Sleep Cycle and Sleep as Android (66). Both apps require the user to place the device on the sleep surface thereby allowing for the detection of movement. The Sleep Cycle application features a “smart alarm” that seeks to wake the user during a preset time range in the morning when it determines a “light sleep” epoch.

The Sleep Cycle application was validated against PSG with a sample of subjects between the ages of 2 and 14 years old (67). The study, conducted by Patel et al., demonstrated no significant relationship for the app measured TST, SL, or SE relative to PSG. Analysis of the data showed a random localization of the data without systematic bias. Additionally, the sleep stage classification from the app had no relationship with the sleep stages as measured by PSG. The Sleep Cycle application is not considered to be useful as a clinical tool due to these findings.

Bhat et al. conducted a validation study on the Sleep Time application (Azumio Inc.) (68). The application significantly overestimated SL compared to PSG. In EBE analysis, the application showed an accuracy of 85.9%, sensitivity of 89.9%, and specificity of 50.0%. For individual sleep stages, the device showed an accuracy of 50% for wake, 54.5% for N1, 33.0% for N2, 71.2% for N3, and 50.6% for REM.

Based on the lack of data validating these approaches to sleep/wake measurement, additional validation of these mobile technologies is needed.

### Cardioballistic Sensors

Cardioballistic sensors measure the body's recoil in response to contraction of the left ventricle of the heart into the aortic arch (69). This process is known as the Cardioballistic effect and is recorded by a ballistocardiograph (BCG). A BCG records the oscillations pertaining to each heartbeat and varies in magnitude depending on the level of cardiac output. These sensors are also capable of recording respiratory activity, body movement, and relative positioning. As such, these devices are suitable for their application in objective sleep measurement. Brink et al. designed a sensor that was installed under each of the four posts of the bed and allowed for the measurement of data which was utilized for sleep/wake scoring.

Beddit is a sensor strip to be placed under the mattress and records data pertaining to body, breathing, and heart movement to calculate relevant sleep variables (70). Tuominen et al. conducted a validation study on the Beddit device to compare its measurement capacity relative to PSG (71). The device underestimated WASO while overestimating TST and SE. The device inherently classifies sleep into “light sleep” (PSG N1+N2) and “deep sleep” (PSG N3). The agreement between PSG and the Beddit device was 42.1% for wake, 55.6% for “light sleep,” and 37.5% for “deep sleep.” Notably, the device does not attempt to identify/classify REM sleep. For individual sleep stages, the agreement between PSG and the Beddit device was 42.1% for wake, 9.6% for N1, 49.6% for N2, and 37.5% for N3.

### Beside Sensors

Schade et al. conducted a validation study on the S+ device (ResMed) compared to PSG (72). S+ is a non-contact sleep monitor using radiofrequency waves to detect movement in bed. The device overestimated TST and underestimated WASO, relative to PSG. In EBE analysis, the device has accuracy of around 87%, sensitivity >90%, and specificity around 70%. For individual sleep stages, accuracy around 64% for light sleep, accuracy around 60% for Stage N1 sleep, accuracy around 65% for Stage N2 sleep, accuracy between 52 and 61% depending on the algorithm used, and accuracy around 61% for REM sleep.

The S+ device was later developed into the SleepScore Max device. SleepScore Max (SleepScore Labs) collects movement data from ultra-wideband radar and information on ambient lighting and room temperature (73). Chinoy et al. investigated the SleepScore Max device (SleepScore Labs) as compared to PSG (58). The device significantly overestimated TST, SE, and SL and underestimated WASO, relative to PSG. In EBE analysis, the device had an accuracy of 88%, sensitivity of 94%, and specificity of 50%. For individual sleep stages, SleepScore Max had an accuracy of 64% for light sleep and an 84% accuracy for both deep and REM sleep.

Toften et al. investigated the validity of the Somnofy device (VitalThings) as compared to PSG (74). Somnofy collects movement and respiration data from an impulse radio ultra-wideband (IR-UWB) radar sensor. Additionally, the device collects information from the sleeping environment such as: light intensity, audible noise, room temperature, air quality, air pressure, and air humidity, using built-in sensors. Sleep summary estimates including TST, WASO, SE, and SL were consistent between Somnofy and PSG. In EBE analysis, the device had an accuracy of 75%, sensitivity of 97%, and specificity of 72%. For individual sleep stages, Somnofy had an accuracy of 75% in detecting Stage N1/N2 sleep, accuracy of 74% in detecting Stage N3 sleep, and 78% accuracy in detecting REM sleep.

### In-bed Sensors

In-bed sensors offer consumers the ability of non-contact sleep measurement and assessment (75). Typically, these devices have sensors that are placed on or under the bed and sometimes into the mattress itself.

Emfit Bed Sensor is a system of foil electrodes placed underneath a mattress which has the capacity to measure movement, respiration, and HR data (76). Kortelainen et al. conducted a validation on the Emfit device compared to PSG. For individual sleep stages, the system had an accuracy of 81% for wake, 75% accuracy for NREM, and 80% accuracy for REM with a kappa of 0.44.

EarlySense is a non-contact sensor paired with a smartphone that collects information on HR, HRV, RR interval, RR variability, and movement to score sleep/wake (77). Tal et al. conducted a validation study of the EarlySense device compared to PSG. The device had good agreement with PSG for TST. In EBE analysis, the device showed an accuracy of 90.5%, sensitivity of 92.5%, and specificity of 80.4%. For individual sleep stages, the agreement between EarlySense and PSG was 80.4% for wake, 64.9% for light sleep (N1+N2), 56.2% for SWS, and 53.7% for REM.

### Wearable/Portable EEG Devices

The rising interest in accurate sleep staging has led to emerging technologies in wearable EEG devices. These devices measure changes in brain waves to mimic the standard scalp EEG that is utilized during a PSG sleep study.

A study conducted by Nakamura et al. investigated the potential of an in-ear EEG device (78). The device could be worn continuously and would reduce burden on participants using the device. In the study, the device was compared directly against standard PSG to measure its overall agreement. The group demonstrated a 74.1% accuracy for five sleep stage classification (wakefulness, NREM1, NREM2, NREM3, and REM) with a kappa score of 0.61.

Another category of emerging technology is EEG headbands that can be worn to also measure changes in cortical activity. A study conducted by Arnal et al. investigated the Dreem headband (79). The study used 25 participants who completed a sleep study with both PSG and the Dreem headband. The data captured by the headband was scored automatically as well as hand scored. The overall agreement for five sleep stage classification (wake, N1, N2, N3, REM) was 83.5% for automatic scoring and 86.4% for hand scoring with kappa scores of 0.748 and 0.798, respectively. The greatest accuracy was achieved for REM sleep and the lowest accuracy for N1 sleep.

Based on the findings of these two studies, wearable EEG devices present themselves as potential alternatives for objective sleep measurement and a reliable tool for measuring the various sleep stages.

### Comparisons to Wearable Actigraphy Devices

Other categories of consumer sleep technology, including phone-based accelerometers, Cardioballistic sensors, bedside sensors, and in-bed sensors, have a few notable strengths and weaknesses compared to the consumer wearables discussed earlier. For strengths, these devices are generally easy for individuals to use with no need to remember to charge or wear the device. This is particularly relevant for the bedside and in-bed sensors. Additionally, these devices have demonstrated similar validation performance to consumer wearables regarding accuracy to PSG. Regarding general weaknesses, these devices tend to be less accurate for individuals who share a bed with a partner or a pet (80). These types of devices detect movement within a particular space, and thus register partner and pet movements. Additionally, the angle of measurement is important and alterations will lead to these devices becoming less accurate.

**Figure 6:**
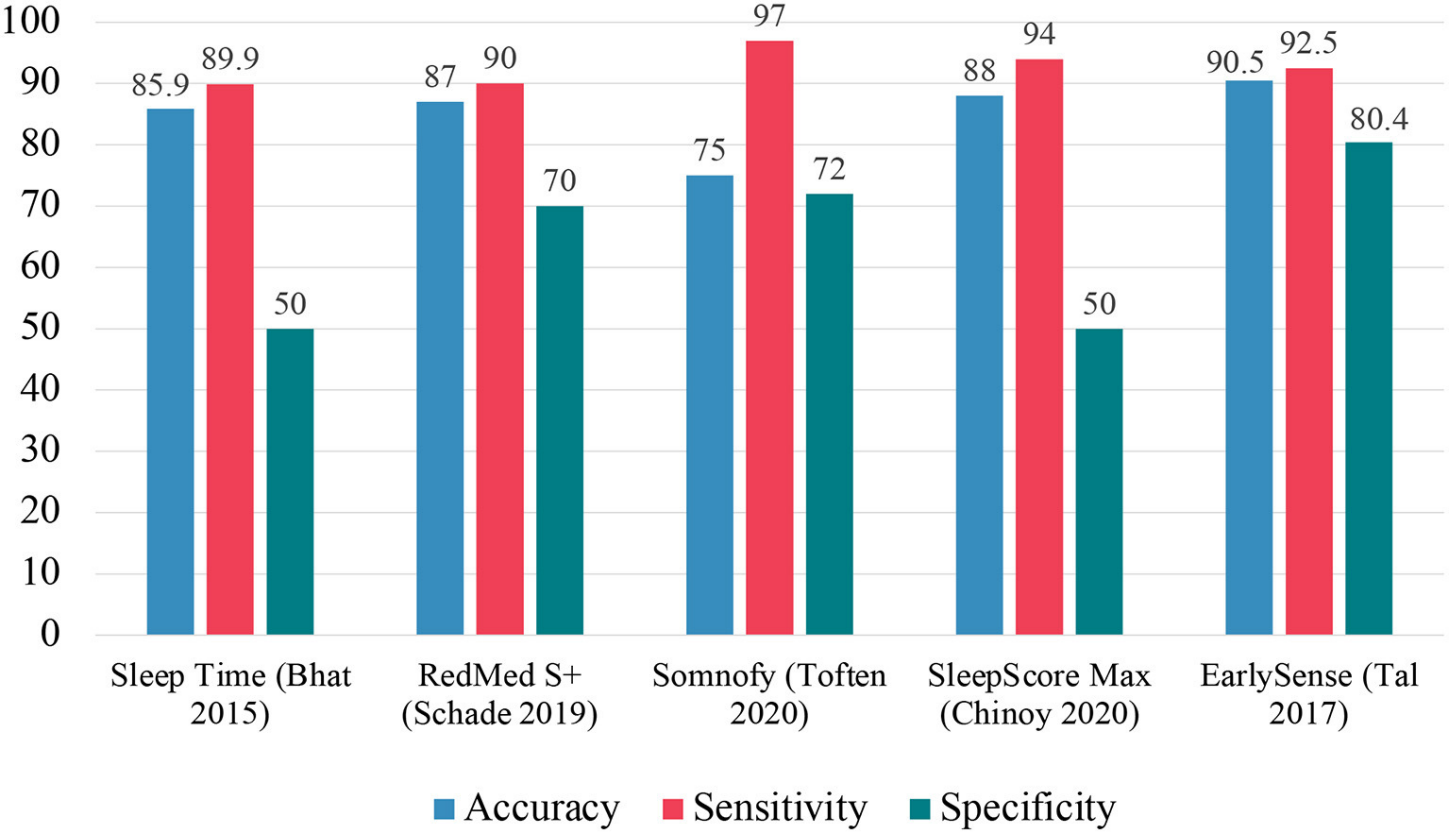
Summary of EBE analysis for other consumer sleep technologies.

Figure 6 summarizes the EBE analyses for the various consumer sleep technologies. In contrast to the data shown in Figure 5 for wearable devices, these data show greater specificity for these devices. One plausible reason for this difference is that nearable devices, such as bedside sensors *via* infrared monitoring, record movements that would not reach the activity threshold for wearable devices (72, 74). These bedside sensors can capture all types of movements and therefore generate greater specificity agreement compared to PSG.

## Applications of Actigraphy

When considering the rich information that is captured regarding an individual's physiology, it is no surprise that these devices are often leveraged in scientific research. Actigraphy devices, as an objective sleep measurement tool, offer the ability to monitor changes in sleep variables in several different settings.

### Epidemiologic Studies

Actigraphs can be used for longitudinal measurements of sleep for pattern generation and correlations with other variables such as cognitive function and risk of cardiometabolic disease.

A study conducted by Blackwell et al. tested the hypothesis that poor sleep, measured objectively by actigraphy, is associated with lower cognition in older women (81). The data examined came from the Study of Osteoporotic Fractures and participants were recorded using the SleepWatch-O, which is a standard piezoelectric actigraph. Cognitive function was assessed with the Mini-Mental State Examination (MMSE) and the Trail Making B Test (Trails B). Results of the study showed that all the measured sleep variables were significantly associated with scores on the MMSE and all, but total sleep time were associated with scores on Trails B.

A study conducted by Stone et al. examined the relationship between actigraphic measurement of sleep duration and fragmentation with risk of recurrent falls in older women (82). Like the study conducted by Blackwell et al., this group utilized data from the Study of Osteoporotic Fractures. The information on falls was collected *via* contact of the participants every 4 months during the study. Results of the study showed that both sleep duration of 5 h or less and sleep duration more than 8 h per night significantly increased the risk of falls. Additionally, the researchers showed that WASO >120 min also increased the likelihood of falls.

A study conducted by Lim et al. aimed to show that sleep fragmentation in older adults is associated with the risk for Alzheimer's disease and the rate of cognitive decline (83). The data utilized for analysis came from the Rush Memory and Aging Project (MAP) and the actigraphic device implemented was the Actical (Philips Respironics). The participants underwent a whole host of cognitive tests to measure global functioning. Results of the study showed that objectively measured sleep fragmentation was associated with higher risk for development of Alzheimer's disease.

A study conducted by Baron et al. aimed to investigate measures of sleep variability, assessed by wrist actigraphy, and its correlation with risk for cardiometabolic disease (84). Participants underwent a 7-day assessment during which they wore the AW-64 device to objectively measure their sleep. Cardiometabolic disease was measured *via* body mass index (BMI), fasting glucose, fasting insulin, glycosylated hemoglobin, c-reactive protein (CRP), and cortisol levels. Results of the study showed a significant association between higher glycosylated hemoglobin and BMI with greater variability in sleep.

### Across the Lifespan

Actigraphy proves useful in the examination of newborns and infants because of the invasiveness of PSG and lack of establishment for its use (85). Sadeh et al. demonstrated that actigraphy has validity in studying this population with overall agreement rates between 88 and 89%. The application of actigraphy can track the development of newborn children and monitor potential maturation deficits. These findings were also confirmed by So et al. with the utilization of actigraphy to study the changes in sleep time throughout early development (86). Notably, the group was able to show that TST starts out at around 60% of the 24-h day at 1 month of age, decreases to 50–55% by 3 months of age, and remains at that level until 12 months of age. The group also showed no sex differences in these trends between male and female infants.

As children continue to age, there is a marked decrease in the amount of daytime sleep (87). Acebo et al. determined this mediated sleep time changes rather than changes in the amount of nocturnal sleep timing. The group utilized actigraphic records of sleep-wake patterns to derive these trends.

By the time children are “school-age” (second, fourth, and sixth grades), actigraphic measurement shows a delay in the onset of sleep and overall decrease in total sleep time (88). Sadeh et al. also demonstrated a high degree of sleep fragmentation present in this age group. Additionally, it was shown that females have great motionless sleep and more time sleep time in general, thus indicating significant sex differences.

These sex differences continue into the adolescent stage as shown by actigraphic measurement of males and females in ninth and tenth grades (89). Carskadon et al. investigated the sleep effects due to change of school start time of students transitioning into tenth grade. The group demonstrated that the earlier wake-up period coincided with a decrease in the TST and increased daytime sleepiness. The use of actigraphy in this context shines light on changes in biology occurring during this phase of life such as circadian rhythm shifts.

### In Behavioral Interventions

Cognitive behavioral therapy for insomnia (CBT-I) is a therapeutic approach aimed at replacing negative behaviors and cognitions surrounding sleep practices with those that will help promote sleep (90). For these types of interventions, the standard is to use sleep diaries which subjectively record sleep, but the inclusion of actigraphy may complement these types of treatments.

In a study conducted by Brooks et al., actigraphy was utilized to measure the effects of sleep treatments within a group of elderly individuals with insomnia (91). Sleep restriction therapy was utilized in this study and led to decreased SL and WASO and increased SE. The researchers highlighted that due to the sensitivity displayed by the actigraph, it is sufficient to demonstrate changes in sleep, which counters any biased estimate of sleep. Any degree of over or underestimation of sleep is likely held constant allowing for the actigraph to be reliable in this context.

Like the study conducted by Brooks et al. and Friedman et al. looked at the effects of sleep restriction therapy as treatment for insomnia in older individuals (92). The group used actigraphic measurement throughout the treatment for all subjects and included PSG measurement in a subgroup of participants. The study highlighted that the actigraphic TST measured was highly correlated with the same TST as measured by PSG.

### For Circadian Research

Circadian rhythmicity is a vital component in the context of sleep vs. wake timing. As such, actigraphy offers the opportunity for objective measurement of sleep to investigate this physiologic driving force.

Teicher collated a review of the use of activity monitoring in individuals with psychiatric disorders such as depression and attention-deficit hyperactivity disorder (ADHD) to show how variance in activity may allude details on circadian dysregulation (93). For instance, the severity of depression correlates with low levels of daytime activity and increases in daytime activity have been shown in those undergoing treatment (94). Additionally, those with ADHD show elevated daytime activity levels and ambulatory monitoring with these devices, especially for children, reinforces an accurate diagnosis and treatment. With 24-h monitoring, researchers can detect any alterations to circadian rhythms that might underly a particular condition.

Siegmund et al. conducted a study of habituation of Tauwema village inhabitants to assess the effects of social zeitgebers and familial synchronization (95). The participants underwent 7 consecutive days of actigraphy measurement. The researchers found that the sleep/wake timing of the adults monitored coincided with the natural light-dark cycle as indicated by decreased activity levels in the nighttime hours. Siegmund et al. also focused on the impact of social factors aligning the sleep/wake timing within families. This phenomenon was visualized *via* the actigraphy data collected. Actigraphic sleep measurement did not impede the participants of the study in any major way, allowed for raw measurement in this population, and subsequently a look into circadian dynamics.

In a study conducted by Pollak et al., actigraphy was utilized with concurrent measurement with PSG to demonstrate the circadian timing of sleep and wake patterns (96). The participants were monitored for 7 days and isolated from temporal cues, allowing for better indication of circadian drive. The actigraphy recordings demonstrated effectiveness by high activity measured during wake and low activity measured during sleep. Notably, the lower activity corresponded with “deep sleep” as measured by PSG. The researchers concluded that actigraphy successfully predicted wake during night periods and accurately measured the circadian cycle length. Despite the observed overestimation of TST and SE, the use of actigraphy was useful to measure circadian effects on sleep and wake in this context.

In a recent study by Cheng et al., circadian rhythm misalignment predictions were made using data collected by wrist actigraphs (97). The study employed a group of 45 shift workers to test the ability of actigraphy to predict dim light melatonin onset (DLMO). DLMO was assessed in a laboratory concurrently with mathematical predictions of this circadian variable. Agreement between in-lab DLMO and actigraphy-predicted DLMO showed a Lin's concordance coefficient of 0.70. The study is significant as the first to indicate the ability of actigraphy to effectively estimate circadian timing as a suitable replacement clinically from in-lab DLMO.

## Limitations in Actigraphic Sleep Measurement

Actigraphy, like other techniques, remains an indirect measure of sleep and thus is subject to its limitations. These devices rely on measuring changes in movement to correlate with changes in sleep vs. wake which presents real issues with sleep fragmentation associated with clinical disorders.

### Difficulties With Insomnia

Insomnia is often categorized as a state of hyperarousal that effects both the central and peripheral nervous systems (98). As a result of this hyperarousal, an individual is unable to enter various sleep stages and remains awake. When an individual is awake but not moving, the actigraphic device will likely incorrectly score this epoch as being sleep when in fact it was “quiet wakefulness” (99). This phenomenon is often found in individuals with complaints of insomnia.

In a study conducted by Hauri et al., 36 individuals with severe complaints of insomnia were monitored with PSG and actigraphy assessments for three nights in a sleep laboratory environment (100). Over the course of the study, the actigraphy measured sleep time was on average 49 min different as compared to PSG. In general, the actigraph overestimated TST and especially in patients with severe insomnia. The researchers proposed that due to these measured differences that actigraphy can be used as an additional tool but should be paired with at least 1 night of PSG measurement.

A more modern study assessed the validity of a consumer device, the Fitbit Flex (an older Fitbit device that did not include PPG), and its capacity to objectively measure sleep in patients with insomnia (101). The accuracy was assessed in conjunction with both actigraphy and PSG measurement with comparisons to a population of “good sleepers.” In good sleepers, TST was overestimated by 6.5 min and SE was overestimated by 1.75% by the Fitbit device as compared to PSG. In comparison, the insomnia group saw TST overestimated by 32.9 min and SE overestimated by 7.9%. Because of these results, the researchers proposed caution in using consumer sleep trackers as a proxy for PSG for clinical and research purposes in insomnia.

### Difficulties With Sleep Staging

Sleep stages were derived using EEG waveforms measured with PSG. As such, standard actigraphy, which generally only uses movement data for sleep staging calculations, fails to be an accurate measure (96). Therefore, developers have utilized the technology in PPG and other physiologic sensors to improve these classifications.

A study conducted by Fonseca et al. aimed to investigate the accuracy that wrist worn actigraphy combined with PPG measurement has in determining individual sleep stages (102). Compared to PSG, the PPG measurement led to an accuracy of 91.5% for wake, 65.7% for N1+N2, 82.5% for N3, 75.3% for NREM, and 78.9% for REM. These findings suggested reliability in utilizing this technique to improve sleep stage scoring compared to PSG.

In the previously mentioned validation study conducted by Beattie et al. the possibility to accurately score the various sleep stages with a consumer device was investigated (57). The Fitbit Surge device tested demonstrated a 69% agreement with PSG measured “light” sleep, a 62% agreement with PSG measured “deep” sleep, and a 72% agreement with PSG measured REM sleep. These results were promising given the utility for inexpensive, reliable measurement of sleep stages.

**Figure 7:**
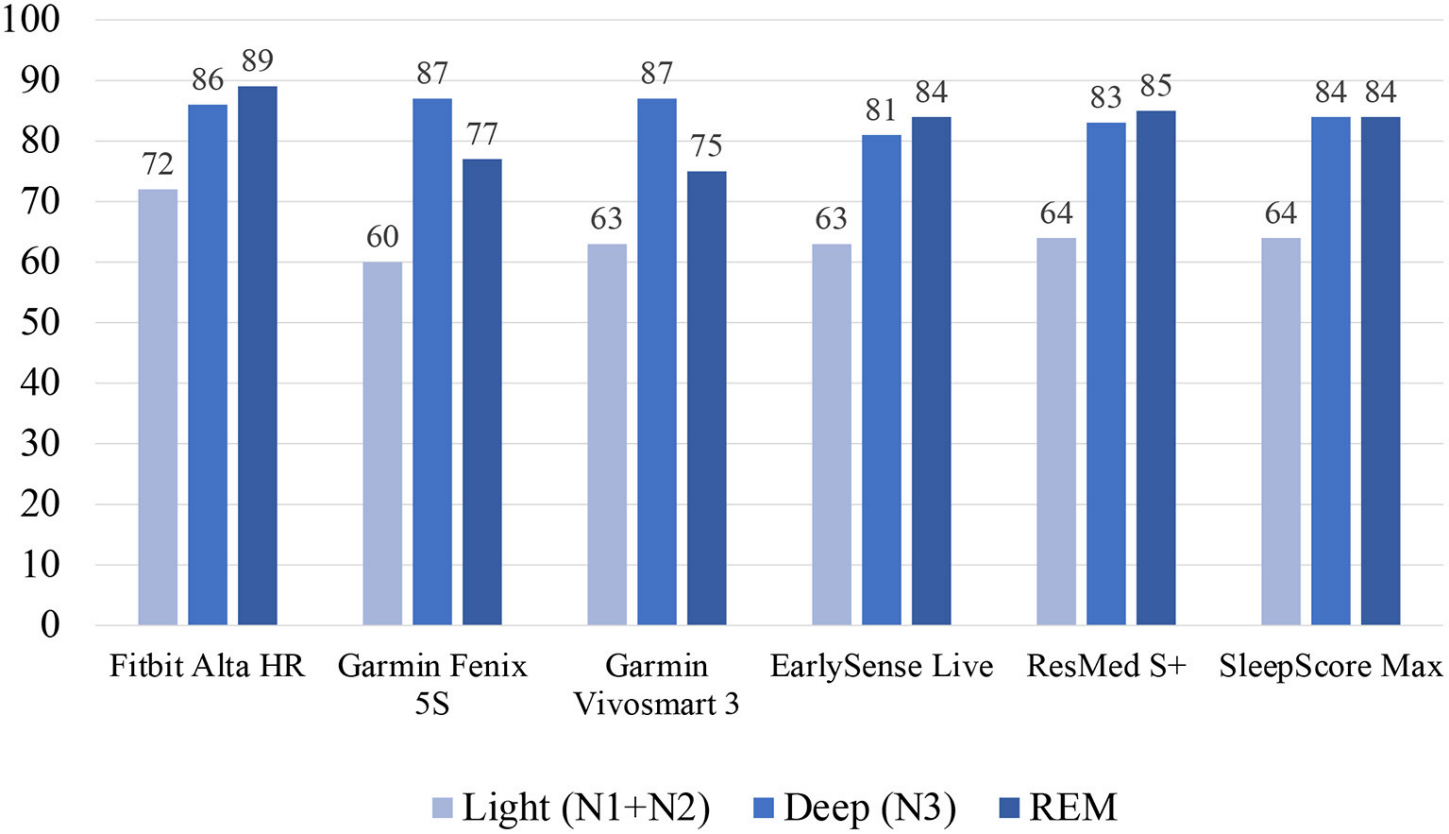
Sleep staging validation for wearables and non-wearables from Chinoy et al. (58).

Additionally, in the previously mentioned paper published by Chinoy et al. several different wearables and non-wearables were studied against PSG for their accuracies of classifying the individual sleep stages (58). The Fitbit Alta HR had accuracies of 72, 86, 89% for light, deep, and REM sleep, respectively. The Garmin Fenix 5S had accuracies of 60, 87, 77% for light, deep, and REM sleep, respectively. The Garmin Vivosmart 3 had accuracies of 63, 87, 75% for light, deep, and REM sleep, respectively. The EarlySense Live nearable device had accuracies of 63, 81, 84% for light, deep, and REM sleep, respectively. The ResMed S+ nearable device had accuracies of 64, 83, 85% for light, deep, and REM sleep, respectively. And the SleepScore Max nearable device had accuracies of 64, 84, 84% for light, deep, and REM sleep, respectively. See Figure 7 for a summary of these data.

These consumer devices, while not perfect substitutes for PSG measurement, prove to be capable of roughly estimating sleep stages. In the previously cited study by Zhai et al. these devices are more capable of accuracy measuring 3 sleep stages (wake, NREM, REM) vs. 4 sleep stages (wake, REM, light, deep) and 5 sleep stages (wake, REM, N3, N2, N1) (64). This study showed 78.2% accuracy for 3 sleep stages, 71.2% for 4 sleep stages, and 65.4% for 5 sleep stages.

### Difficulties With Sleep Apnea and Sleep Fragmentation

Like individuals with insomnia, sleep apnea and other disorders effectively fragment sleep due to arousals over the course of the night. These arousals impact the scoring ability of actigraphy devices and thus considerations need to be taken into account when using these devices in scientific research (103).

Middelkoop et al. investigated the combined use of wrist actigraphy and self-assessment measures in the screening process for obstructive sleep apnea (OSA) with additional use of respiratory measurement (104). The study utilized a sample of 116 subjects that were suspected to have OSA. Results of the study showed that high apnea index (AI) scores were associated with both self-reported sleep disturbances and increased activity as measured by actigraphy. The only measure that significantly correlated with high AI scores was the duration of immobility periods. Because of this, the researchers concluded that objective sleep measurement with actigraphy fails to reliably identify those with OSA due to its poor specificity for wake episodes.

A potential avenue to improve objective sleep measurement in individuals with OSA is *via* application of multisensory consumer devices. One such study that investigated this practicality was conducted by Moreno-Pino et al. with usage of a Fitbit device (105). The study utilized individuals with OSA alongside Fitbit and PSG assessment. Results of the study were able to confirm the diagnosis of OSA in 55 out of 65 (84.6%) participants. The researchers concluded that consumer wearables still have insufficient accuracy for use in clinical settings but that optimizing features, such as wake detection (specificity), could potentially make them more capable for use in clinical populations.

### Limitations With Different Types of Skin

A problem specific to PPG technology utilized with consumer wearables is variability across different types of skin. In the study conducted by Bent et al. variance tended to be more pronounced during periods of activity as opposed to periods of rest (106). The largest trend was variance across different devices indicating that some devices may be more accurate than others for larger populations of people. Notably, the Apple Watch had the lowest error across all studied groups. But the investigators found no statistically significant difference across skin tones. The results of this study were, however, argued against by an editorial published in SLEEP by Colvonen et al. The authors indicated that the study included a very small sample size including only nine individuals with the darkest of pigments (107). As such, this topic warrants further consideration and devices should be validated amongst diverse populations. This is especially important given the growing literature addressing sleep health disparities (108, 109).

In addition to variability in natural skin tone, tattoos may also pose a problem for this technology. Although peer-reviewed research on this topic is yet unavailable, a popular press report on the Apple Watch found that the device experienced difficulties accurately measuring heart rate in individuals with tattoos (110). The ink from tattoos may interfere with the ability of PPG sensors to accurately measure HR. Apple has since modified the hardware of their devices but continue to claim on their website that individuals with tattoos will see errors in accurate HR detection (111).

## Future Directions

Given the performance of multisensory, wearable sleep devices compared to standard actigraphy devices, the field of clinical sleep research should continue to utilize these power pieces of technology for the goal of measuring as well as improving overall sleep health. Future studies are needed to further improve these devices both in terms of their validity as well as their recording metrics.

### Concept of Validation

In 2019, Depner et al. reported on the results of an international consensus conference on best practices for validation studies (112). There are three major steps that were identified for this process: (1) validation study implementation, (2) statistical analysis and reporting, and (3) validation study outcomes. Specifically, the document notes that careful consideration needs to be taken when designing a new validation study including a necessity for direct comparison to PSG and appropriate statistical analyses, such as Bland-Altman plots and Epoch by Epoch analysis. The group advocated for following these “best practices” to prioritize standardization and replication. A device that is validated once needs to be repeatedly validated in numerous populations.

This publication raises the notion of reconsidering the concept of validation. In this context, validation is not an event, rather validation is a process (113). Devices need to be continuously and rigorously tested to determine their accuracy in measuring sleep objectively. These points were directly addressed by both Meghini et al. as well as a related commentary by Goldstein and Depner (114, 115). The argument was postulated that the weight is shifted from validity of devices to the performance of devices. By placing more weight on the performance of devices, a user can determine if a certain device fits the needs of an individual or the goal of a research study. With this shift, it is likely that consumer devices will get better footing in the realm of research as they typically perform as well or better than existing research-grade actigraphs.

### Ceiling Effect in Accuracy

Despite numerous algorithms, hardware and software innovations, wearable devices are unable to exactly replicate findings of gold-standard PSG. This may be due to a ceiling effect in accuracy against this standard. For consumer devices to quantify sleep, peripheral changes in physiology are measured and interpreted, and these interpretations are meant to correlate with interpretations of measures used to quantify changes within the brain. These correlations are possible because of the interconnected pathway between brain changes and peripheral changes (116). But as EEG is an indirect measure of brain activity, PPG is an indirect measure of heart activity. Aspects of physiology are lost in translation between these two pathways thereby indicating a limitation in the degree to which the measures can exactly translate to each other. To combat this problem, machine learning and AI might prove useful but likely new metrics will need to be generated and discovered that make sleep and wake prediction more reliable. Still, peripheral signals may never be able to exactly approximate sleep stages. Perhaps future directions can better explore what aspects of the sleep experience peripheral signals can explain, which are otherwise difficult to quantify using PSG or self-report measures (113). For example, since PSG is unable to approximate habitual patterns and self-report is unable to approximate arousals, perhaps wearables (optimized to do so) may provide the most useful data available on objective arousals in habitual sleep.

### New Sensing Approaches and Novel Biomarkers for Estimating Sleep/Wake

**Figure 8:**
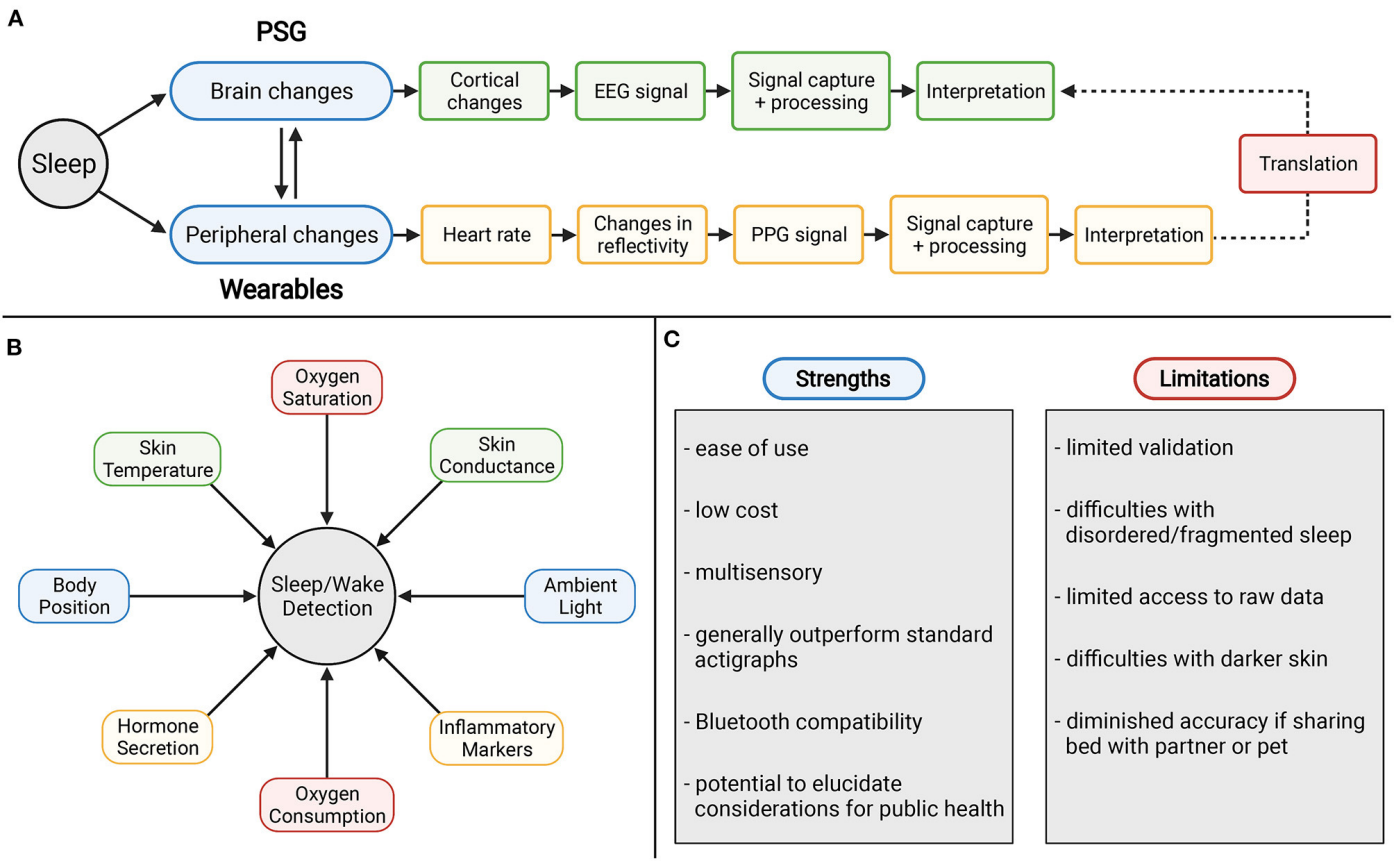
**(A)** Ceiling effect in accuracy with wearable devices using PPG technology. **(B)** Potential novel metrics for sleep and wake measurement. **(C)** Strengths and weaknesses of consumer sleep technology.

New potential variables will need to consider how physiologic changes coincide with sleep (see Figure 8B for potential metrics). As wearables are typically located at the wrist, new metrics and signals ought to be easily measured at the periphery, but underly important changes in physiology that occur during sleep. The combination of multiple signals offers the potential for more successful AI and machine learning techniques to improve sleep staging and scoring.

#### Oxygen Saturation and Consumption

Oxygen saturation has traditionally been included in standard polysomnography with pulse oximetry methods. This technique allows for detection of hypopnea events that are associated with physiological arousals due to sleep apnea *via* drops in arterial oxygen saturation (117). Continuous recording of oxygen saturation may elucidate limitations of actigraphic measurement of sleep apnea as well as overall improve specificity.

Additionally, measurement of oxygen consumption (VO2) across the night offers similar potential. In a study of eight normal male subjects, VO2 was collected over the course of 28 subject-nights (118). The data reflected an initial decrease in VO2 followed by a rise in VO2 in the morning. The researchers also investigated the magnitude of VO2 associated with sleep stages across the night. These data showed that VO2 during wake and stage 1 sleep were significantly higher than other stages of sleep, REM sleep significantly lower than stage 2, and stage 3 and 4 were not significantly different from either each other or REM and stage 2 sleep. With future studies and utilizing modern devices, these observed trends offer improved scoring for not just sleep vs. wake but also for individual sleep stages as well.

#### Skin Conductance and Temperature

Changes in skin conductance *via* measurement of electrodermal activity (EDA) has been characterized over the years as it has implications for autonomic nervous system activity. Most peaks of EDA activity have been demonstrated to occur during stage 2 and stage 3/4 sleep (119). These high frequency patterns of EDA have been termed “storms” with amplitudes highest in slow wave sleep (SWS). More recently, EDA recording was tested against the gold standard PSG as a method for ambulatory sleep monitoring (120). The EDA sensor was worn at the wrist and measured changes in skin conductance across the sleep measurement period using the parameters of electrodermal activity-smoothed feature (EDASEF). The study showed significant differences in EDASEF between wake and stage 1, wake and stage 2, wake and stage 3/4, and wake and REM. Based on these findings, the researchers built a scoring algorithm for sleep/wake which yielded an accuracy of 86%, sensitivity of 97%, and specificity of 75%. With these findings substantiate this method as a valid metric for improving scoring.

The 24-h rhythm of body temperature has been well-documented, with redistribution of heat causing a drop in core body temperature (121, 122). The drop in core body temperature coincides with increases in temperature at the periphery including both proximal and distal skin. In a study conducted by Kräuchi et al. it was found that the increased temperature at the extremities, including hands and feet, predicted rapid sleep onset (123). Regarding sleep stages themselves, REM sleep is marked by increases in sympathetic activation and thus vasoconstriction whereas NREM sleep is associated with parasympathetic dominance and therefore vasodilation. As a novel metric, future studies are needed to investigate if the observed changes in heat as well as the autonomic activity within sleep stages themselves offer implications for improved scoring.

#### Ambient Light and Body Position

Since some of the earliest actigraphy devices, photometers have been included to record light exposure over the 24-h day (124). This pattern is generally utilized in scoring to help define the major sleep period (9). However, these sensors have not normally been translated over the multisensory devices for utilization in sleep scoring. Devices such as the Apple Watch do include ambient light sensors for auto-adjustment for the brightness of the display, but these data are not utilized otherwise. In the validation study for the Apple Watch conducted by Walch et al. the group demonstrated that a light proxy by using the accessible accelerometry data drastically improved scoring (60). Because of the implications of ambient light in assessment of circadian rhythms, including this metric could elucidate limitations in these devices automatic sleep detection and improve sleep latency measurement.

Another strategy to potentially improve these devices is with measurement of body position and changes that occur across the night period. This measurement will likely improve sleep/wake measurement for those with sleep disorders such as periodic limb movement disorder (PLMD). These associations were investigated amongst 11 subjects and hypothesized that adverse body positions resulting in PLMD episodes due to spinal cord affect or declining body tissue perfusion (125). Changes in body position are often recorded during PSG measurement and inclusion within multisensory wearables may offer similar value and extrapolate use of these devices for those suffering from sleep disorders.

#### Inflammatory Markers and Hormone Secretion

Cortisol impacts many aspects of physiology including glucose mobilization and immune responses. The hormone has been demonstrated to have a diurnal rhythm and as such is a prominent signal for wakefulness. Reliable measurement of cortisol levels at the periphery, in any capacity, may dramatically improve the utility of devices to determine wakefulness due to these trends. Additionally, cortisol elevations have been associated with both acute partial and total sleep deprivation (126). After periods of sleep deprivation, plasma cortisol was elevated the following evening thus delaying cortisol level recovery and disrupting the normal diurnal rhythm. Other hormones that have been correlated with circadian rhythms include growth hormone, melatonin, leptin, and ghrelin (127). Like cortisol, measurement of these hormones captures the circadian rhythm and can be extrapolated for improved scoring as well as utilized for identifying disruptions in their normal secretion.

These contributions would similarly be improved with measures of inflammation *via* recording changes in interleukin 6 (IL-6) and CRP. These markers of inflammation yield assessment of insufficient sleep and can improve the application of multisensory devices for their use in epidemiologic studies and measurement of cardiometabolic risk. IL-6 and CRP have been demonstrated to be elevated amongst short sleepers (128). A recent study conducted by Jagannath et al. demonstrated the potential for monitoring interleukin-1β and CRP in individuals with inflammatory bowel disease (129). The wearable device was able to measure these biomarkers by analyzing sweat released by eccrine sweat glands. These findings have yet to be demonstrated in sleep but the ability to assessment systemic inflammation by multisensory wearables may be useful in the context of sleep research for improving sleep/wake detection.

## Conclusions

From its inception, actigraphy has proven to be a useful tool for objective sleep measurement. Due to the implementation of additional sensors, such as PPG, newer devices offer the potential for improved quantification of sleep parameters. Many of these devices are available to the general public, even though they have demonstrated accuracy similar to devices more traditionally seen as scientific devices (113). Despite the limitations of this technology, the sleep science community should continue to invest resources into improvement of these devices for the rich information they can provide on individuals overall health.

Figure 8C summarizes some strengths and limitations for consumer devices. For strengths, wearable devices have a relatively ease of use and low cost. As multisensory devices they are tools that generally outperform accepted standard actigraphs. With Bluetooth compatibility they offer immediate access to data which offers potential to elucidate considerations for public health. These devices are not without limitations, however. Some of these include limited validation, difficulties with sleep disorders, limited access to raw data, difficulties with darker skin, and diminished accuracy if sharing the bed with a partner or pet. However, with addressing some of the key limitations and future directions in this field, consumer wearable and non-wearable devices can readily break the stigma against their use with shifting the focus to addressing their performance in context rather than validity.

## Author Contributions

ML and MG conceptualized the paper, drafted the outline of the document, refined the outline, and edited the final document. ML wrote the first draft of the manuscript and generated the figures. All authors contributed to manuscript revision, read, and approved the submitted version.

## Conflict of Interest

MG reports grants in the past 12 months from Jazz Pharmaceuticals, Kemin Foods, and CeraZ Technologies. He has received consulting fees from Fitbit, Casper, Athleta, Natrol, and Idorsia. The remaining authors declare that the research was conducted in the absence of any commercial or financial relationships that could be construed as a potential conflict of interest.

## Publisher's Note

All claims expressed in this article are solely those of the authors and do not necessarily represent those of their affiliated organizations, or those of the publisher, the editors and the reviewers. Any product that may be evaluated in this article, or claim that may be made by its manufacturer, is not guaranteed or endorsed by the publisher.
